# Putative Candidate Drug Targets for Sarcopenia-Related Traits Identified Through Mendelian Randomization Analysis of the Blood Proteome

**DOI:** 10.3389/fgene.2022.923429

**Published:** 2022-07-22

**Authors:** Bin-Bin Chen, Jia-Qi Wang, Xiang-He Meng, Zhe Luo, Xiao-Wen Liu, Hui Shen, Hong-Mei Xiao, Hong-Wen Deng

**Affiliations:** ^1^ Center for System Biology, Data Sciences and Reproductive Health, School of Basic Medical Science, Central South University, Changsha, China; ^2^ Tulane Center of Biomedical Informatics and Genomics, Deming Department of Medicine, School of Medicine, Tulane University School, New Orleans, LA, United States

**Keywords:** sarcopenia, proteomics, GWAS, genetics, Mendelian randomization analysis

## Abstract

**Purpose:** The increasing prevalence of sarcopenia remains an ongoing challenge to health care systems worldwide. The lack of treatments encouraged the discovery of human proteomes to find potential therapeutic targets. As one of the major components of the human proteome, plasma proteins are functionally connected with various organs of the body to regulate biological processes and mediate overall homeostasis, which makes it crucial in various complex processes such as aging and chronic diseases. By performing a systematic causal analysis of the plasma proteome, we attempt to reveal the etiological mechanism and discover drug targets for sarcopenia.

**Methods:** By using data from four genome-wide association studies for blood proteins and the UK Biobank data for sarcopenia-related traits, we applied two-sample Mendelian randomization (MR) analysis to evaluate 310 plasma proteins as possible causal mediators of sarcopenia-related traits: appendicular lean mass (ALM) and handgrip strength (right and left). Then we performed a two-sample bidirectional Mendelian randomization analysis for the identified putatively causal proteins to assess potential reverse causality that the trait values may influence protein levels. Finally, we performed phenome-wide MR analysis of the identified putatively causal proteins for 784 diseases to test the possible side effects of these proteins on other diseases.

**Results:** Five plasma proteins were identified as putatively causal mediators of sarcopenia-related traits. Specifically, leukocyte immunoglobulin-like receptor subfamily B member 2 (LILRB2), asporin (ASPN), and contactin-2 (CNTN2) had potential causal effects on appendicular lean mass, and ecto-ADP-ribosyltransferase 4 (ART4) and superoxide dismutase 2 (SOD2) had putative causal effects on the handgrip strength, respectively. None of the five putatively causal proteins had a reverse causality relationship with sarcopenia-related traits, and no side effects on other diseases were identified.

**Conclusion:** We identified five plasma proteins that may serve as putatively potential novel drug targets for sarcopenia. Our study attested to the value of two-sample MR analysis in identifying and prioritizing putatively potential therapeutic targets for complex diseases.

## Introduction

Sarcopenia is defined as progressive skeletal muscle disorder. Its prominent clinical manifestations are known as muscle mass loss and function declining with aging (Cruz-Jentoft et al., 2010; Liguori et al., 2018). It is a major contributor to frailty, dysfunction in older people, poor health-related quality of life, and the peril of premature death, thus putting a great burden on the health care system (Cruz-Jentoft and Sayer, 2019). Sarcopenia was officially recognized as a muscle disorder with the diagnosis code ICD-10-MC in 2016 (Anker et al., 2016). Several working groups of sarcopenia have included both low muscle mass (e.g., appendicular lean mass, ALM) and declined muscle function/strength (e.g., handgrip strength) in their definitions although cutoffs for different components may vary (Cruz-Jentoft et al., 2014; Cruz-Jentoft et al., 2019; Chen et al., 2020). More importantly, there has been no effective pharmacological therapy for sarcopenia (Gonzalez-Freire et al., 2017). It is urgent and essential to explore the pathogenesis of sarcopenia and identify new drug targets. It has been estimated that by selecting candidate therapeutic targets with genetic evidences, the success rate of selected targets can be doubled in subsequent clinical trial (Nelson et al., 2015).

Plasma proteins are known to play a critical role in biological processes such as transport, signal transduction, growth, repair and defense against infection (Geyer et al., 2016; Corbo et al., 2021). They provide a window into the pathophysiological status of humans (Zheng et al., 2020; Elhadad et al., 2021). However, there are only sporadic data of the association between plasma proteins and muscle mass (Han et al., 2021; Huemer et al., 2021), and no studies have explored the relationship between muscle strength and plasma proteins. Recently, with the development of new techniques for high-throughput protein quantitation, genome-wide association studies (GWAS) have been able to simultaneously reveal the genetic determinants of thousands of blood proteins (Suhre et al., 2021).

Mendelian randomization (MR) is a statistical method that uses genetic variants as instrumental variables (IVs) to estimate the causal relationship between the exposure (for example, proteins) and the outcome (for example, handgrip strength). MR analysis in combination with proteomics has identified novel biomarkers and potential drug targets for many complex diseases (Chong et al., 2019; Liu et al., 2019; Gudmundsdottir et al., 2020; Zheng et al., 2020). For example, MR was applied in a hypothesis-driven manner to assess the causality between selected biomarkers and stroke risk including C–C motif chemokine ligand 2(CCL2), Chitinase-3–like protein 1 (CHI3L1), C-reactive protein (CRP), cystatin C (CST3), apolipoprotein (a) (LPA), matrix metalloproteinase-12 (MMP12), and proprotein convertase subtilisin/Kexin type 9 (PCSK9) (Chong et al., 2019). In addition, MR analysis can also assess the potential side effects of a drug target and reveal its adverse effect (Chong et al., 2019; Gudmundsdottir et al., 2020; Zheng et al., 2020).

In the present study, we attempted to integrate genetic and proteomic data through MR analysis to identify new effective drug targets for sarcopenia. We firstly performed a meta-analysis on the association results from the currently available data of four GWAS to obtain genetic variants that are strongly associated with 3,576 plasma proteins (Ahola-Olli et al., 2017; Suhre et al., 2017; Sun et al., 2018; Folkersen et al., 2020). Next, we applied MR analysis to evaluate the causal relationships between sarcopenia-related traits (i.e., ALM and handgrip strength) and proteins. After that, bidirectional MR analysis was performed on the identified putatively causal proteins and assess potential reverse causality. Finally, we predicted target-mediated side-effects for the identified putatively causal proteins by phenome-wide analysis of 784 disease characteristics (Zhou et al., 2018; Chong et al., 2019).

## Methods

### Obtaining Genetic Determinants of Plasma Protein Levels

We firstly identified a set of genetic variants that were significantly associated with the blood protein levels (Emdin et al., 2017). As in previous studies (Chong et al., 2019; Zheng et al., 2020), to accomplish this, we combined four recently published and currently available proteomics GWAS data: INTERVAL (*n* = 3,301) (Sun et al., 2018), KORA F4/QMDiab (Cooperative Health Research in the Region of Augsburg/Qatar Metabolomics Study on Diabetes) (*n* = 1,335) (Suhre et al., 2017), YFS/FINRISK 2002 (The Cardiovascular Risk in Young Finns Study/the levels of chronic disease risk factors in Finland) (*n* = 8,332) (Ahola-Olli et al., 2017), and Olink CVD-I (Summary statistics from GWAS of Olink CVD-I proteins in the SCALLOP consortium) (*n* = 30,931) (Folkersen et al., 2020).

The four studies which included all the currently publicly available datasets, all focused on Europeans, used high-throughput and quantitative technologies to quantify blood samples for proteins. INTERVAL (Sun et al., 2018) analyzed 2,994 proteins via SOMAScan assay, a multiplexed aptamer-based immunoassay. Log transformed protein levels were then adjusted for linear regression based on age, sex, duration between blood draw and processing (binary, ≤ 1 day/> 1 day) and the first three major components of ancestry derived from multi-dimensional scales. KORA F4/QMDiab (Suhre et al., 2017) analyzed 1,124 proteins via the SOMAScan assay. KORA F4 (Suhre et al., 2017) used PLINK to fit linear models to inverse-normalized probe levels, using age, gender, and body mass index as covariates. In QMDiab (Suhre et al., 2017) linear regression models were fitted using PLINK with age, sex, body mass index, diabetes state, the first three principal components of the genotype data, and the first three principal components of the proteomics data as covariates. In YFS/FINRISK 2002 (Ahola-Olli et al., 2017), a total of 48 cytokines were measured by using Bio-Rad’s premixed Bio-Plex Pro Human Cytokine 27-plex Assay and 21-plex Assay, and Bio-Plex 200 reader with Bio-Plex 6.0 software. Cytokine distributions were first normalized with inverse transformation. Then, by calculating residuals of a linear regression model, the transformed phenotypes were adjusted for age, sex, body mass index, and the top ten genetic principal components (Ahola-Olli et al., 2017). Olink CVD-I (Folkersen et al., 2020) analyzed 90 proteins using modified antibodies conjugated to oligonucleotides. Genetic analyses were done by using additive model regressions to adjust population structure and study-specific parameters.

The four published proteomic data included a total of 3,576 proteins, 882 of which were subjected to the genome-wide association analyses in two or more of the four studies (Ahola-Olli et al., 2017; Suhre et al., 2017; Sun et al., 2018; Folkersen et al., 2020). We performed a meta-analysis of those proteins present in two or more studies with Genome-Wide Association Meta-analysis (GWAMA) software using an inverse variance weighted model with fixed effect (Mägi and Morris, 2010; Baselmans et al., 2019; McGuire et al., 2021). All single nucleotide polymorphisms (SNPs) that were strongly (*p* < 5 × 10^−8^) and independently (linkage disequilibrium, LD, *R* ^2^ < 0.001) associated with exposures (i.e., each protein) were selected as instrumental variables (IVs). Subsequently, to ensure the specificity of IVs, SNPs associated with more than three proteins were removed. Exposures associated with fewer than three independent IVs were also excluded. When there was no SNP in the outcome data, the proxy SNP (*R* ^2^ > 0.9) of LD links was used. A total of 310 proteins with valid IVs were assessed in the following MR analyses.

### GWAS Summary Statistics of Sarcopenia-Related Traits

We used the GWAS summary-level data of ALM and handgrip strength (left and right) to explore the causal relationships between plasma proteins and sarcopenia (Cruz-Jentoft et al., 2019). The GWAS data of sarcopenia-related traits from the UK Biobank study was obtained from the MR-Base database (Hemani et al., 2018). Briefly, ALM was measured using bioelectrical impedance analysis (BIA) (Sergi et al., 2017), and handgrip strength was measured by a calibrated hydraulic hand dynamometer adjusting hand size (Sudlow et al., 2015). We used absolute grip strength rather than relative handgrip strength (absolute handgrip strength/weight) as a proxy for muscle strength, because the correlation of absolute handgrip strength and muscle strength may be higher than the relative grip strength (Wind et al., 2010). The GWAS study of ALM (*n* = 450,243) was adjusted for age, age^2^, sex, assessment center, genotyping array, appendicular fat mass (AFM), and the first ten genetic principal components (Pei et al., 2020). The GWAS study of handgrip strength (kg) was adjusted for age, age^2^, sex, sex × age, and sex × age^2^ (Bahat et al., 2016; Kitamura et al., 2021). The genetic associations of handgrip strength (left hand, *n* = 461,026; right hand, *n* = 461,089) were from the Integrative Epidemiology Unit (IEU) analysis of UK Biobank phenotypes.

### Statistical Analysis

We mainly used the inverse variance weighted (IVW) method for MR analysis to infer the causal relationship between plasma proteins and sarcopenia-related traits (Burgess et al., 2013). The causal effect *β* was estimated as $\frac{\sum_{i}{\text{α}}_{i}{\text{γ}}_{i}{\text{σ}}_{i}^{-2}}{\sum_{i}{\text{γ}}_{i}^{2}{\text{σ}}_{i}^{-2}}$ , where *α*_*i*_ was defined as the association effect of the *i*th IV on plasma protein, *γ*_*i*_ was the association effect of the *i*th IV on sarcopenia with standard error *σ*_*i*_ (Bull et al., 2020).

To make valid causal inferences, MR analysis must satisfy three hypotheses: 1) that the genetic variants (IVs) were strongly associated with the exposure, 2) the genetic variants were not associated with any of the confounders associated with the exposure and the outcome, 3) the genetic variants did not affect the outcome unless it may be achieved through an association with the exposure (i.e., no horizontal pleiotropy) (Emdin et al., 2017; Zhao et al., 2020). To ensure the robustness of the results, we also used the following additional analyses. The weighted median MR approach is robust in that it can produce correct estimates even when up to 50% of SNPs are invalid IVs (Qian et al., 2020). And we used MR-Egger regression and MR-Egger with a simulation extrapolation (SIMEX) to evaluate whether there is a horizontal pleiotropic effect (Bowden et al., 2015; Bowden et al., 2016a; Sekula et al., 2016; Vermeulen et al., 2021). The heterogeneity test used I^2^ in the IVW method to test whether there is heterogeneity among the causal effects obtained by IVs alone compared with the randomly expected value (Augusteijn et al., 2019). Finally, the “leave-one-out” method was adopted to be sensitivity analysis in this study. The “leave-one-out” method is to re-analyze the results after removing single SNP one by one, to judge the influence of each SNP on the results and evaluate the stability of the results (Huan et al., 2019).

Bidirectional MR was used to assess whether there was a feedback loop between sarcopenia-related traits and causal risk proteins, which could cause false-positive results difficult to interpret (Yang et al., 2022). We assessed reverse causality between five putative causal proteins as the outcome variables and sarcopenia-related traits as the exposure by MR analysis with the IVW method (Burgess et al., 2013; Holmes et al., 2017). Sensitivity analysis was performed using MR-Egger, and weighted median MR (Bowden et al., 2016a; Bowden et al., 2016b).

A Bonferroni-corrected *p* value threshold was used, which takes the numbers of tested plasma proteins into account (*p* value = 0.05/310 = 1.65 × 10^−4^). As the Bonferroni correction was considered to be conservative and the ALM and handgrip strength were genetic related traits, we reported proteins passed this threshold for each trait rather than the study as a whole. All MR analyses were completed using the ‘TwosampleMR’ package version 0.5.5 in R version 4.0.3 (Hemani et al., 2018). Because all the data sets for the study were downloaded from the public domain, written informed consent and ethical approval were not required (Yang et al., 2022).

### Phenome-Wide MR Analysis of 784 Disease Traits

We performed phenome-wide MR analysis to identify the causality between the putatively causal proteins and other disease traits. The primary purpose of the phenome-wide MR analysis was to assess potential side effects (beneficial or adverse) associated with hypothetical interventions to reduce the risk of sarcopenia by targeting the putatively causal plasma proteins. The IVs were the same as those previously selected to identify the causal proteins for sarcopenia-related traits. The association analysis of disease outcomes was conducted using publicly available data from the UK Biobank cohort (N ≤ 408,961). Diseases in the cohort were defined by PheCodes. PheCodes is a system for organizing the international classification codes for diseases and related health problems into phenotypic results suitable for the characteristics of a wide range of disease phenotypes of genetic analysis (Denny et al., 2013; Zhou et al., 2018). The disease definition scheme combines the ICD-9 codes of a hospital into hierarchical PheCodes, with each PheCode representing one more or less specific disease group. As in previous studies (Chong et al., 2019), we excluded disease results generated from fewer than 500 cases due to a lack of statistical validity. In total, a total of 784 disease characteristics were selected for analysis, including 16 categories, as detailed in Supplemental Table S1.

## Results

**FIGURE 1:**
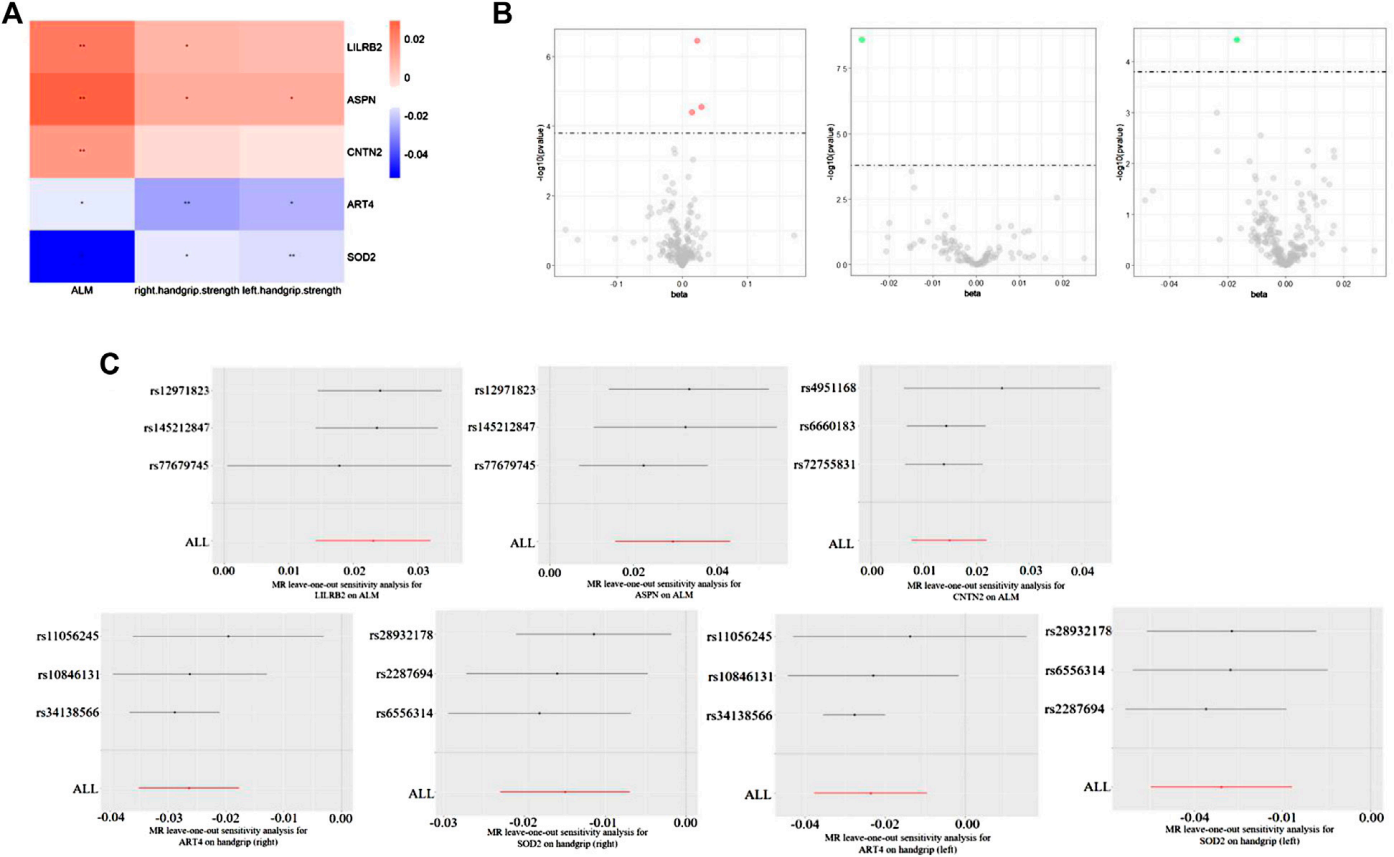
**(A)** Heat map of Sarcopenia-related traits (rows) and the five plasma proteins (columns). The colour scale runs from blue (negative correlation) to red (positive correlation). Note: *, *p* < 0.05, **, *p* < 0.05/310. **(B)** Volcano maps of Sarcopenia-related traits and the five plasma proteins. **(C)** MR leave-one-out sensitivity analysis for the five plasma proteins on ALM and handgrip (left and right).

The four recently published proteomics GWAS summary data sets (see methods) were combined for a Meta-analysis using an inverse variance weighted model with a fixed effect. We presented the results of the meta GWAS analysis for the proteomics used in this paper, such as the *p* value of Q statistic in Tables 1–Tables 5. Meta-analysis was performed on LILRB2, CNTN2 and SOD2 of five putative causal proteins, and the results of meta-analysis showed no heterogeneity among SNPS (Tables 1, 3, 5). After removing proteins presented in less than two studies, 310 plasma proteins with the numbers of IV ≥ 3 (Burgess et al., 2013), were included in the following MR analyses to identify the potential causality between plasma proteins and sarcopenia-related traits. And we presented statistically significant IVs for putative causal proteins in Tables 1–Tables 1. The IVs of proteins used were all trans-regulated, i.e., the SNPs were > 1 Mb away from the transcription start sites of their respective associated genes. At Bonferroni significance (Bonferroni *p* = 0.05/310 = 1.65 × 10^–4^), five proteins showed putatively causal effects on sarcopenia-related traits (Table 6; Figures 1A,B).

**TABLE 1:** Correlation of genetic IVs to LILRB2 and ALM.

SNPs	LILRB2							ALM		
	Beta	SE	*p* value	reference_allele	other_allele	q_*p*-value	I^2^	Beta	SE	*p* value
rs12971823	0.26	0.034	3.27 × 10^−14^	A	G	1	NaN	0.0044	0.0031	0.15
rs145212847	0.45	0.082	2.75 × 10^−8^	T	C	1	NaN	0.0086	0.0060	0.16
rs77679745	−0.62	0.042	1.56 × 10^−50^	T	C	1	NaN	−0.016	0.0033	2.30 × 10^−6^

Note: IVs, Instrumental variables; LILRB2, Leukocyte immunoglobulin-like receptor subfamily B member 2; ALM, appendicular lean mass; SNPs, Single nucleotide polymorphism; SE, standard error.

**TABLE 2:** Correlation of genetic IVs to ASPN and ALM.

SNPs	ASPN							ALM		
	Beta	SE	*p* value	reference_allele	other_allele	q_*p*-value	I^2^	Beta	SE	*p* value
rs148794554	−0.79	0.10	7.77 × 10^−15^	T	C	—	—	−0.019	0.0085	0.028
rs545515434	-0.45	0.066	7.12 × 10^−12^	T	G	—	—	−0.020	0.0050	9.50 × 10^−5^
rs62564371	−0.30	0.047	1.79 × 10^−10^	A	G	—	—	−0.0062	0.0034	0.071

Note: IVs, Instrumental variables; ASPN, asporin; ALM, appendicular lean mass; SNPs, Single nucleotide polymorphism; SE, standard error.

**TABLE 3:** Correlation of genetic IVs to CNTN2 and ALM.

SNPs	CNTN2							ALM		
	Beta	SE	*p* value	reference_allele	other_allele	q_*p*-value	I^2^	Beta	SE	*p* value
rs4951168	−0.71	0.030	8.34 × 10^−128^	T	C	1	NaN	−0.0094	0.0028	6.70 × 10^−4^
rs6660183	0.44	0.076	4.82 × 10^−9^	T	C	1	NaN	0.010	0.0061	0.099
rs72755831	0.49	0.085	9.03 × 10^−9^	T	C	1	NaN	0.013	0.0064	0.039

Note: IVs, Instrumental variables. CNTN2, contactin-2. ALM, appendicular lean mass; SNPs, Single nucleotide polymorphism; SE, standard error.

**TABLE 4:** Correlation of Genetic IVs to ART4 and Handgrip (right and left).

SNPs	ART4							Handgrip (right)			Handgrip (left)		
	Beta	SE	*p* value	reference_allele	other_allele	q_*p*-value	I^2^	Beta	SE	*p* value	Beta	SE	*p* value
rs10846131	0.16	0.025	1.29 × 10^−10^	A	G	—	—	−0.0044	0.0015	0.0036	−0.0045	0.0015	0.0033
rs11056245	−0.36	0.026	1 × 10^−200^	T	C	—	—	0.011	0.0016	3.30 × 10^−11^	0.010	0.0016	2.90 × 10^−10^
rs34138566	0.21	0.036	1.00 × 10^−8^	A	C	—	—	−0.0022	0.0021	0.30	0.00047	0.0021	0.82

Note: IVs, Instrumental variables; ART4, ecto-adp-ribosyltransferase 4; SNPs, Single nucleotide polymorphism; SE, standard error.

**TABLE 5:** Correlation of Genetic IVs to SOD2 and Handgrip (right and left).

SNPs	SOD2							Handgrip (right)			Handgrip (left)		
	Beta	SE	*p* value	reference_allele	other_allele	q_*p*-value	I^2^	Beta	SE	*p* value	Beta	SE	*p* value
rs2287694	0.27	0.041	3.54 × 10^−11^	T	C	1	NaN	−0.0031	0.0024	0.18	−0.0030	0.0024	0.21
rs28932178	0.28	0.030	2.19 × 10^−21^	T	C	1	NaN	−0.0066	0.0021	0.0020	−0.0056	0.0021	0.0086
rs6556314	0.27	0.026	2.66 × 10^−24^	T	C	1	NaN	−0.0031	0.0016	0.057	−0.0049	0.0016	0.0025

Note: IVs, Instrumental variables; SOD2, Superoxide dismutase 2; SNPs, Single nucleotide polymorphism; SE, standard error.

**TABLE 6:** Association between plasma proteins and sarcopenia-related traits using Weighted median and Inverse variance weighted.

Exposures	Outcomes	Number of IVs	Weighted median		Inverse variance weighted	
			Beta(95% CI)	*p* value	beta (95% CI)	*p* value
LILRB2	ALM	3	0.023 (0.013, 0.033)	2.93 × 10^−6^	0.023 (0.014, 0.032)	3.54 × 10^−7^
ASPN	ALM	3	0.024 (0.0062, 0.041)	7.76 × 10^−3^	0.029 (0.016, 0.043)	2.82 × 10^−5^
CNTN2	ALM	3	0.015 (0.0071, 0.022)	1.19 × 10^−4^	0.015 (0.0078, 0.022)	4.04 × 10^−5^
ART4	Handgrip strength (right)	3	-0.028 (-0.037, -0.020)	5.27 × 10^−12^	-0.026 (-0.035, -0.018)	2.5 × 10^−9^
SOD2	Handgrip strength (right)	3	-0.011 (-0.021, -0.0021)	1.59 × 10^−2^	-0.015 (-0.023, -0.0069)	2.76 × 10^−4^
ART4	Handgrip strength (left)	3	-0.028 (-0.036, -0.019)	3.76 × 10^−10^	-0.023 (-0.038, -0.0096)	9.95 × 10^−4^
SOD2	Handgrip strength (left)	3	-0.018 (-0.027, -0.0087)	1.54 × 10^−4^	-0.017 (-0.025, -0.0089)	3.72 × 10^−5^

Note: IVs, Instrumental variables; CI, confidence interval; LILRB2, Leukocyte immunoglobulin-like receptor subfamily B member 2; ASPN, asporin; CNTN2, contactin-2; ART4, ecto-adp-ribosyltransferase 4; SOD2, Superoxide dismutase 2; ALM, appendicular lean mass.

Specifically, higher expression of leukocyte immunoglobulin-like receptor subfamily B member 2 (LILRB2) (effect size = 0.023, *p* value = 3.54 × 10^−7^), asporin (ASPN) (effect size = 0.029, *p* value = 2.82 × 10^−5^) and contactin-2 (CNTN2) (effect size = 0.015, *p* value = 4.04 × 10^−5^) may cause increased ALM, and ecto-adp-ribosyltransferase 4 (ART4) (effect size = -0.026, *p* value = 2.51 × 10^−9^) and Superoxide dismutase 2 (SOD2) (effect size = -0.017, *p* value = 3.72 × 10^−5^) may cause decreased right and left handgrip strength, respectively. In addition, ART4 (*p* value = 9.95 × 10^−4^) and SOD2 (*p* value = 2.76 × 10^−4^) also exhibited nominal significance for left and right handgrip strength, respectively. The MR-Egger regression and MR-Egger using SIMEX results showed that the estimated values for the intercept terms were null for all the five identified proteins and sarcopenia-related traits (Table 7), suggesting that horizontal pleiotropy did not significantly influence the results (Table 7). The “leave-one-out” sensitivity analyses were shown in Figure 1C respectively. After SNP is successively two-sample MR removed, SNPs will not have a substantial influence on the results, indicating that two-sample MR analysis results are robust and reliable (Figure 1C).

**TABLE 7:** Association between plasma proteins and sarcopenia-related traits using MR Egger and MR Egger using SIMEX.

Exposures	Outcomes	Number of IVs	MR Egger				MR Egger using SIMEX				I^2^ ^(IVW)^
			Intercept (95% CI)	*p* value	beta (95% CI)	*p* value	Intercept (95% CI)	*p* value	Beta slope (95% CI)	*p* value	
LILRB2	ALM	3	-0.0037 (-0.015, 0.0076)	0.64	0.030 (0.0062, 0.055)	0.25	-0.0048	Na	0.032	Na	0
ASPN	ALM	3	-0.011 (-0.036, 0.014)	0.47	0.047 (-0.019, 0.11)	0.30	-0.0046 (-0.026, 0.017)	0.71	0.033 (-0.010, 0.077)	0.27	16%
CNTN2	ALM	3	-0.0073 (-0.023, 0.0090)	0.44	0.026 (-0.0021, 0.053)	0.17	-0.0094 (-0.032, 0.013)	0.47	0.034 (-0.0086, 0.077)	0.22	0
ART4	Handgrip strength (right)	3	0.0016 (-0.0053, 0.0086)	0.69	-0.034 (-0.061, -0.0069)	0.13	0.0029 (-0.0060, 0.012)	0.59	-0.038 (-0.074, -0.0017)	0.18	73%
SOD2	Handgrip strength (right)	3	0.056 (-0.032, 0.15)	0.43	-0.22 (-0.55, 0.10)	0.41	0.18	Na	-0.67	Na	0
ART4	Handgrip strength (left)	3	0.0021 (-0.0090, 0.013)	0.75	-0.033 (-0.077, 0.0096)	0.27	0.0041 (-0.010, 0.018)	0.63	-0.040 (-0.097, 0.018)	0.31	30%
SOD2	Handgrip strength (left)	3	0.0098 (-0.079, 0.098)	0.86	-0.053 (-0.38, 0.27)	0.80	0.054 (-0.16, 0.27)	0.71	-0.21 (-0.95, 0.53)	0.38	0

Note: IVs, Instrumental variables; CI, confidence interval; LILRB2, Leukocyte immunoglobulin-like receptor subfamily B member 2; ASPN, asporin; CNTN2, contactin-2; ART4, ecto-adp-ribosyltransferase 4; SOD2, Superoxide dismutase 2; ALM, appendicular lean mass.

Bidirectional MR analyses (Bonferroni *p* = 0.05/5 = 0.01) found that sarcopenia-related traits had no causal effect on the identified proteins (Table 8). The results showed that the causal effects of five causal proteins on sarcopenia-related traits were reliable with no evidence of reverse causality.

**TABLE 8:** Mendelian randomization estimates of sarcopenia-related traits on plasma proteins.

Exposures	Outcomes	Number of IVs	MR Egger				Weighted median		Inverse variance weighted	
			intercept (95% CI)	*p* value	beta (95% CI)	*p* value	beta (95% CI)	*p* value	beta (95% CI)	*p* value
ALM	LILRB2	527	-0.0023 (-0.0078, 0.0033)	0.42	0.12 (-0.13,0.37)	0.34	0.020 (-0.16, 0.20)	0.84	0.020 (-0.095, 0.13)	0.74
ALM	ASPN	527	0.0013 (-0.0044, 0.0070)	-0.65	-0.13 (-0.39, 0.12)	0.31	-0.15 (-0.33, 0.019)	0.080	-0.14 (-0.25, -0.26)	0.016
ALM	CNTN2	527	0.0013 (-0.0042, 0.0068)	0.63	-0.74 (-0.32, 0.17)	0.55	-0.045 (-0.22, 0.13)	0.62	0.039 (-0.15, 0.075)	0.50
Handgrip strength (right)	ART4	146	0.0028 (-0.0012, 0.069)	0.17	-3.3 (-6.71, 0.20)	0.067	0.16 (-0.45, 0.77)	0.61	-1.1 (-2.2, 0.018)	0.054
Handgrip strength (right)	SOD2	146	0.0030 (-0.013, 0.019)	0.70	-0.41 (-1.7, 0.90)	0.54	-0.32 (-0.92, -0.28)	0.29	-0.22 (-0.61, -0.17)	0.28
Handgrip strength (left)	ART4	131	0.049 (0.0020, 0.095)	0.043	-5.1 (-9.0, -1.1)	0.013	0.074 (-0.53, 0.68)	0.81	-1.3 (-2.5, -0.058)	0.040
Handgrip strength (left)	SOD2	131	0.0058 (-0.011, 0.023)	0.50	-0.51 (-2.0, 0.94)	0.49	-0.24 (-0.90, 0.41)	0.46	-0.0070 (-4.3, 0.41)	0.97

Note: IVs, Instrumental variables; CI, confidence interval; LILRB2, Leukocyte immunoglobulin-like receptor subfamily B member 2; ASPN, asporin; CNTN2, contactin-2; ART4, ecto-adp-ribosyltransferase 4; SOD2, Superoxide dismutase 2; ALM, appendicular lean mass.

After the five plasma proteins were identified to be potentially causally associated with sarcopenia-related traits, phenome-wide MR analysis was performed to comprehensively assess the potential side effects profile of each target. After multiple hypothesis test corrections (*p* ≤ 6.38 × 10^−5^), none of the five sarcopenia-trait-related proteins have significant potential causal relationships with the other 784 disease traits tested (Supplementary Tables S2–S6). In particular, we paid special attention to diseases related to the pathogenesis of sarcopenia, such as skeletal musculoskeletal-related diseases. For example, there are 21 diseases (Supplementary Table S7) with *p* < 0.05 in phenome-wide MR analysis related to LILRB2, and five diseases belonging to skeletal muscle diseases include: polymyalgia rheumatica, spondylosis and allied disorders, malunion and nonunion of fracture, rheumatoid arthritis, and other inflammatory polyarthropathies. The results showed that LILRB2 was related to the musculoskeletal system.

## Discussion

We investigated the putatively potential causal effects of genetically predicted plasma protein levels on sarcopenia-related traits and identified three putatively causal proteins for ALM (LILRB2, ASPN, and CNTN2) and two for handgrip strength (ART4 and SOD2). Higher expression of LILRB2, ASPN and CNTN2 may cause increased ALM, and higher expression of ART4 and SOD2 may cause decreased right and left handgrip strength, respectively. None of the identified proteins has reverse causal effects on sarcopenia-related traits and had no side effects on the other 784 diseases. None of these causal relationships has been reported before, to our knowledge. Our study attested to the value of two-sample MR in identifying and prioritizing putatively potential therapeutic targets for complex diseases.

LILRB2 is the receptor for class I MHC antigens known to be an immune inhibitory receptor to suppress the immune system. In human, LILRB2 recognize a broad spectrum of HLA-A, HLA-B, HLA-C, HLA-G, and HLA-F alleles (Shiroishi et al., 2006). We did not identify a causal effect of LILRB2 on other diseases in this study. But its key role in cancer and Alzheimer’s disease (AD) has been reported in many studies (Kim et al., 2013; Hu et al., 2017). Specifically, in cancer, LILRB2 was reported as a potentially myeloid immune checkpoint that regulates tumor-associated myeloid cells and provokes immune reaction against the development of tumor (Chen et al., 2018). In Alzheimer’s disease, soluble β-amyloid (Aβ) oligomers were known to lead to synaptic loss associated with AD, And human LILRB2 was a β-amyloid receptor, so blocking the function of LILRB2 is a potential therapeutic target for AD, (Kim et al., 2013). The direction of action is opposite to that obtained in this study, so the feasibility and the utility of LILRB2 to be a therapeutic target for sarcopenia may be relatively low.

ASPN is a kind of leucine-rich small proteoglycan (SLRP). A previous study showed that there was a significant reduction of ASPN (*p* value = 0.004) in the extracellular matrix of gastrocnemius muscles in old mice compared with adult mice (Lofaro et al., 2021). We found here that ASPN may causally increase ALM. But the molecular mechanism underlying this causal relationship needs to be further explored.

SOD2 is a manganese-containing enzyme. It is located in mitochondria that protect cells from oxidative stress through scavenging reactive oxygen species (ROS) (Xu et al., 2020). Mitochondrial dysfunction of skeletal muscle is related to the pathogenesis of many diseases, including type 2 diabetes, muscle atrophy, osteoporosis and aging-related sarcopenia (Kalinkovich and Livshits, 2015), since muscle is a highly metabolically active tissue that critically relies on both aerobic and anaerobic metabolic energy supply system especially aerobic metabolic energy supply. . However, as one of the major process of aerobic metabolic energy supply, oxidative phosphorylation produces by-products that potentially damaging radicals such as the superoxide anion (O2.-) (Flynn and Melov, 2013). Superoxide can damage components of the electron transport chain and other cellular constituents, while SOD2 which is a critical enzyme of eukaryotic systems can efficiently convert superoxide to the less reactive hydrogen peroxide (H2O2) and protect the cell from ROS. Therefore, the disfunction of SOD2 can result in numerous pathological phenotypes in metabolically active tissues. A previous study found that in wild-type mice of glycolytic and oxidative gastrocnemius, use of Simvastatin increased mRNA expression of Sod1 and Sod2 genes but decreased grip strength (Panajatovic et al., 2020). Consistent in our study, SOD2 was also identified to be causally associated with decreased handgrip strength.

Only two previous studies have evaluated plasma proteins for association with sarcopenia-related traits (Han et al., 2021; Huemer et al., 2021). Huemer et al. used the machine learning approach in the KORA S4/FF4 study, and determined kallikrein-6, C-C motif chemokine 28 (CCL28), and tissue factor pathway inhibitor are biomarkers for muscle mass and serine protease 27 for fat mass (Huemer et al., 2021). CCL28 and metalloproteinase inhibitor 4 (TIMP4) constitute biomarkers of a combination of low muscle and high-fat mass (Huemer et al., 2021). Han et al. identified the increase in the relative abundance of follistatin (FST) and IGFBP1 are causally associated with the decrease in trunk fat-free mass and whole-body fat-free mass by conducting a two-sample MR analysis based on the GWAS summary statistics of 23 body composition traits and 2,656 plasma proteins (Han et al., 2021). However, these proteins are not included in our study, so we cannot confirm whether there is a causal relationship between these proteins and sarcopenia-related traits.

Despite some novel findings, the present study has some potential limitations. First, the established causality was not replicated in independent samples, and there is a lack of experiments to further verify our conclusions. Therefore, the results await further validation. Secondly, due to the limitation of data availability, most of the study participants included in the current analysis are of European descent. It is necessary to include participants of non-European ancestry in future studies to enable transethnic MR analyses, which are expected to reduce the radical bias and lead to more generalizable findings. Thirdly, it is important to recognize that MR analysis has several other potential limitations. Although a variety of genetic instruments are used to improve the ability of MR, there is still a certain risk of pleiotropy despite extensive sensitivity analysis.

## Data Availability

The original contributions presented in the study are included in the article/Supplementary Material, further inquiries can be directed to the corresponding authors.

## References

[B1] Ahola-Olli A. V. Würtz P. Havulinna A. S. Aalto K. Pitkänen N. Lehtimäki T. (2017). Genome-Wide Association Study Identifies 27 Loci Influencing Concentrations of Circulating Cytokines and Growth Factors. Am. J. Hum. Genet. 100 (1), 40–50. 10.1016/j.ajhg.2016.11.007 27989323 PMC5223028

[B2] Anker S. D. Morley J. E. von Haehling S. (2016). Welcome to the ICD‐10 Code for Sarcopenia. J. Cachexia Sarcopenia Muscle 7 (5), 512–514. 10.1002/jcsm.12147 27891296 PMC5114626

[B3] Augusteijn H. E. M. van Aert R. C. M. van Assen M. A. L. M. (2019). The Effect of Publication Bias on the Q Test and Assessment of Heterogeneity. Psychol. Methods 24 (1), 116–134. 10.1037/met0000197 30489099

[B4] Bahat G. Tufan A. Tufan F. Kilic C. Akpinar T. S. Kose M. (2016). Cut-off Points to Identify Sarcopenia According to European Working Group on Sarcopenia in Older People (EWGSOP) Definition. Clin. Nutr. 35 (6), 1557–1563. 10.1016/j.clnu.2016.02.002 26922142

[B5] Baselmans B. M. L. Jansen R. Jansen R. Ip H. F. van Dongen J. Abdellaoui A. (2019). Multivariate Genome-Wide Analyses of the Well-Being Spectrum. Nat. Genet. 51 (3), 445–451. 10.1038/s41588-018-0320-8 30643256

[B6] Bowden J. Davey Smith G. Burgess S. (2015). Mendelian Randomization with Invalid Instruments: Effect Estimation and Bias Detection through Egger Regression. Int. J. Epidemiol. 44 (2), 512–525. 10.1093/ije/dyv080 26050253 PMC4469799

[B7] Bowden J. Davey Smith G. Haycock P. C. Burgess S. (2016b). Consistent Estimation in Mendelian Randomization with Some Invalid Instruments Using a Weighted Median Estimator. Genet. Epidemiol. 40 (4), 304–314. 10.1002/gepi.21965 27061298 PMC4849733

[B8] Bowden J. Del Greco F. M. Minelli C. Davey Smith G. Sheehan N. A. Thompson J. R. (2016a). Assessing the Suitability of Summary Data for Two-Sample Mendelian Randomization Analyses Using MR-Egger Regression: The Role of the I2 Statistic. Int. J. Epidemiol. 45 (6), 1961–1974. 10.1093/ije/dyw220 27616674 PMC5446088

[B9] Bull C. J. Bell J. A. Murphy N. Sanderson E. Davey Smith G. Timpson N. J. (2020). Adiposity, Metabolites, and Colorectal Cancer Risk: Mendelian Randomization Study. BMC Med. 18 (1), 396. 10.1186/s12916-020-01855-9 33327948 PMC7745469

[B10] Burgess S. Butterworth A. Thompson S. G. (2013). Mendelian Randomization Analysis with Multiple Genetic Variants Using Summarized Data. Genet. Epidemiol. 37 (7), 658–665. 10.1002/gepi.21758 24114802 PMC4377079

[B11] Chen H.-M. van der Touw W. Wang Y. S. Kang K. Mai S. Zhang J. (2018). Blocking Immunoinhibitory Receptor LILRB2 Reprograms Tumor-Associated Myeloid Cells and Promotes Antitumor Immunity. J. Clin. Invest. 128 (12), 5647–5662. 10.1172/jci97570 30352428 PMC6264729

[B12] Chen L.-K. Woo J. Assantachai P. Auyeung T.-W. Chou M.-Y. Iijima K. (2020). Asian Working Group for Sarcopenia: 2019 Consensus Update on Sarcopenia Diagnosis and Treatment. J. Am. Med. Dir. Assoc. 21 (3), 300–307. e302. 10.1016/j.jamda.2019.12.012 32033882

[B13] Chong M. Sjaarda J. Pigeyre M. Mohammadi-Shemirani P. Lali R. Shoamanesh A. (2019). Novel Drug Targets for Ischemic Stroke Identified through Mendelian Randomization Analysis of the Blood Proteome. Circulation 140 (10), 819–830. 10.1161/circulationaha.119.040180 31208196

[B14] Corbo C. Li A. A. Poustchi H. Lee G. Y. Stacks S. Molinaro R. (2021). Analysis of the Human Plasma Proteome Using Multi‐Nanoparticle Protein Corona for Detection of Alzheimer's Disease. Adv. Healthc. Mater. 10 (2), e2000948. 10.1002/adhm.202000948 33169521

[B15] Cruz-Jentoft A. J. Baeyens J. P. Bauer J. M. Boirie Y. Cederholm T. Landi F. (2010). Sarcopenia: European Consensus on Definition and Diagnosis: Report of the European Working Group on Sarcopenia in Older People. Age Ageing 39 (4), 412–423. 10.1093/ageing/afq034 20392703 PMC2886201

[B16] Cruz-Jentoft A. J. Bahat G. Bauer J. Boirie Y. Bruyère O. Cederholm T. (2019). Sarcopenia: Revised European Consensus on Definition and Diagnosis. Age Ageing 48 (1), 16–31. 10.1093/ageing/afy169 30312372 PMC6322506

[B17] Cruz-Jentoft A. J. Landi F. Schneider S. M. Zúñiga C. Arai H. Boirie Y. (2014). Prevalence of and Interventions for Sarcopenia in Ageing Adults: A Systematic Review. Report of the International Sarcopenia Initiative (EWGSOP and IWGS). Age Ageing 43 (6), 748–759. 10.1093/ageing/afu115 25241753 PMC4204661

[B18] Cruz-Jentoft A. J. Sayer A. A. (2019). Sarcopenia. Lancet 393 (10191), 2636–2646. 10.1016/s0140-6736(19)31138-9 31171417

[B19] Denny J. C. Bastarache L. Ritchie M. D. Carroll R. J. Zink R. Mosley J. D. (2013). Systematic Comparison of Phenome-Wide Association Study of Electronic Medical Record Data and Genome-Wide Association Study Data. Nat. Biotechnol. 31 (12), 1102–1111. 10.1038/nbt.2749 24270849 PMC3969265

[B20] Elhadad M. A. Wilson R. Zaghlool S. B. Huth C. Gieger C. Grallert H. (2021). Metabolic Syndrome and the Plasma Proteome: From Association to Causation. Cardiovasc Diabetol. 20 (1), 111. 10.1186/s12933-021-01299-2 34016094 PMC8138979

[B21] Emdin C. A. Khera A. V. Kathiresan S. (2017). Mendelian Randomization. JAMA 318 (19), 1925–1926. 10.1001/jama.2017.17219 29164242

[B22] Flynn J. M. Melov S. (2013). SOD2 in Mitochondrial Dysfunction and Neurodegeneration. Free Radic. Biol. Med. 62, 4–12. 10.1016/j.freeradbiomed.2013.05.027 23727323 PMC3811078

[B23] Folkersen L. Gustafsson S. Wang Q. Hansen D. H. Hedman Å. K. Schork A. (2020). Genomic and Drug Target Evaluation of 90 Cardiovascular Proteins in 30,931 Individuals. Nat. Metab. 2 (10), 1135–1148. 10.1038/s42255-020-00287-2 33067605 PMC7611474

[B24] Geyer P. E. Kulak N. A. Pichler G. Holdt L. M. Teupser D. Mann M. (2016). Plasma Proteome Profiling to Assess Human Health and Disease. Cell Syst. 2 (3), 185–195. 10.1016/j.cels.2016.02.015 27135364

[B25] Gonzalez-Freire M. Semba R. D. Ubaida-Mohien C. Fabbri E. Scalzo P. Højlund K. (2017). The Human Skeletal Muscle Proteome Project: A Reappraisal of the Current Literature. J. Cachexia Sarcopenia Muscle 8 (1), 5–18. 10.1002/jcsm.12121 27897395 PMC5326819

[B26] Gudmundsdottir V. Zaghlool S. B. Emilsson V. Aspelund T. Ilkov M. Gudmundsson E. F. (2020). Circulating Protein Signatures and Causal Candidates for Type 2 Diabetes. Diabetes 69 (8), 1843–1853. 10.2337/db19-1070 32385057 PMC7372075

[B27] Han B.-X. Yan S.-S. Xu Q. Ni J.-J. Wei X.-T. Feng G.-J. (2021). Mendelian Randomization Analysis Reveals Causal Effects of Plasma Proteome on Body Composition Traits. J. Clin. Endocrinol. Metab. 107, e2133–e2140. 10.1210/clinem/dgab911 34922401

[B28] Hemani G. Zheng J. Elsworth B. Wade K. H. Haberland V. Baird D. (2018). The MR-Base Platform Supports Systematic Causal Inference across the Human Phenome. Elife 7, e34408. 10.7554/eLife.34408 29846171 PMC5976434

[B29] Holmes M. V. Ala-Korpela M. Smith G. D. (2017). Mendelian Randomization in Cardiometabolic Disease: Challenges in Evaluating Causality. Nat. Rev. Cardiol. 14 (10), 577–590. 10.1038/nrcardio.2017.78 28569269 PMC5600813

[B30] Hu T. Wang S. Chen C. Sun J. Yang X. (2017). Real-Time Analysis of Binding Events between Different Aβ(1-42) Species and Human Lilrb2 by Dual Polarization Interferometry. Anal. Chem. 89 (4), 2606–2612. 10.1021/acs.analchem.6b04950 28219245

[B31] Huan T. Joehanes R. Song C. Peng F. Guo Y. Mendelson M. (2019). Genome-Wide Identification of DNA Methylation QTLs in Whole Blood Highlights Pathways for Cardiovascular Disease. Nat. Commun. 10 (1), 4267. 10.1038/s41467-019-12228-z 31537805 PMC6753136

[B32] Huemer M. T. Bauer A. Petrera A. Scholz M. Hauck S. M. Drey M. (2021). Proteomic Profiling of Low Muscle and High Fat Mass: A Machine Learning Approach in the KORA S4/FF4 Study. J. Cachexia Sarcopenia Muscle 12 (4), 1011–1023. 10.1002/jcsm.12733 34151535 PMC8350207

[B33] Kalinkovich A. Livshits G. (2015). Sarcopenia--The Search for Emerging Biomarkers. Ageing Res. Rev. 22, 58–71. 10.1016/j.arr.2015.05.001 25962896

[B34] Kim T. Vidal G. S. Djurisic M. William C. M. Birnbaum M. E. Garcia K. C. (2013). Human LilrB2 Is a β-Amyloid Receptor and its Murine Homolog PirB Regulates Synaptic Plasticity in an Alzheimer's Model. Science 341 (6152), 1399–1404. 10.1126/science.1242077 24052308 PMC3853120

[B35] Kitamura A. Seino S. Abe T. Nofuji Y. Yokoyama Y. Amano H. (2021). Sarcopenia: Prevalence, Associated Factors, and the Risk of Mortality and Disability in Japanese Older Adults. J. Cachexia Sarcopenia Muscle 12 (1), 30–38. 10.1002/jcsm.12651 33241660 PMC7890144

[B36] Liguori I. Russo G. Aran L. Bulli G. Curcio F. Della-Morte D. (2018). Sarcopenia: Assessment of Disease Burden and Strategies to Improve Outcomes. Clin. Interv. Aging 13, 913–927. 10.2147/cia.S149232 29785098 PMC5957062

[B37] Liu H.-M. Hu Q. Zhang Q. Su G.-Y. Xiao H.-M. Li B.-Y. (2019). Causal Effects of Genetically Predicted Cardiovascular Risk Factors on Chronic Kidney Disease: A Two-Sample Mendelian Randomization Study. Front. Genet. 10, 415. 10.3389/fgene.2019.00415 31130989 PMC6509563

[B38] Lofaro F. D. Cisterna B. Lacavalla M. A. Boschi F. Malatesta M. Quaglino D. (2021). Age-Related Changes in the Matrisome of the Mouse Skeletal Muscle. Int. J. Mol. Sci. 22 (19), 10564. 10.3390/ijms221910564 34638903 PMC8508832

[B39] Mägi R. Morris A. P. (2010). GWAMA: Software for Genome-wide Association Meta-Analysis. BMC Bioinforma. 11, 288. 10.1186/1471-2105-11-288 PMC2893603 20509871

[B40] McGuire D. Jiang Y. Liu M. Weissenkampen J. D. Eckert S. Yang L. (2021). Model-Based Assessment of Replicability for Genome-Wide Association Meta-Analysis. Nat. Commun. 12 (1), 1964. 10.1038/s41467-021-21226-z 33785739 PMC8009871

[B41] Nelson M. R. Tipney H. Painter J. L. Shen J. Nicoletti P. Shen Y. (2015). The Support of Human Genetic Evidence for Approved Drug Indications. Nat. Genet. 47 (8), 856–860. 10.1038/ng.3314 26121088

[B42] Panajatovic M. V. Singh F. Roos N. J. Duthaler U. Handschin C. Krähenbühl S. (2020). PGC‐1α Plays a Pivotal Role in Simvastatin‐Induced Exercise Impairment in Mice. Acta Physiol. 228 (4), e13402. 10.1111/apha.13402 PMC7100017 31605661

[B43] Pei Y.-F. Liu Y.-Z. Yang X.-L. Zhang H. Feng G.-J. Wei X.-T. (2020). The Genetic Architecture of Appendicular Lean Mass Characterized by Association Analysis in the UK Biobank Study. Commun. Biol. 3 (1), 608. 10.1038/s42003-020-01334-0 33097823 PMC7585446

[B44] Qian Y. Ye D. Huang H. Wu D. J. H. Zhuang Y. Jiang X. (2020). Coffee Consumption and Risk of Stroke: A Mendelian Randomization Study. Ann. Neurol. 87 (4), 525–532. 10.1002/ana.25693 32034791

[B45] Sekula P. Del Greco M. F. Pattaro C. Köttgen A. (2016). Mendelian Randomization as an Approach to Assess Causality Using Observational Data. J. Am. Soc. Nephrol. 27 (11), 3253–3265. 10.1681/asn.2016010098 27486138 PMC5084898

[B46] Sergi G. De Rui M. Stubbs B. Veronese N. Manzato E. (2017). Measurement of Lean Body Mass Using Bioelectrical Impedance Analysis: A Consideration of the Pros and Cons. Aging Clin. Exp. Res. 29 (4), 591–597. 10.1007/s40520-016-0622-6 27568020

[B47] Shiroishi M. Kuroki K. Rasubala L. Tsumoto K. Kumagai I. Kurimoto E. (2006). Structural Basis for Recognition of the Nonclassical MHC Molecule HLA-G by the Leukocyte Ig-Like Receptor B2 (LILRB2/LIR2/ILT4/CD85d). Proc. Natl. Acad. Sci. U. S. A. 103 (44), 16412–16417. 10.1073/pnas.0605228103 17056715 PMC1637596

[B48] Sudlow C. Gallacher J. Allen N. Beral V. Burton P. Danesh J. (2015). UK Biobank: An Open Access Resource for Identifying the Causes of a Wide Range of Complex Diseases of Middle and Old Age. PLoS Med. 12 (3), e1001779. 10.1371/journal.pmed.1001779 25826379 PMC4380465

[B49] Suhre K. Arnold M. Bhagwat A. M. Cotton R. J. Engelke R. Raffler J. (2017). Connecting Genetic Risk to Disease End Points through the Human Blood Plasma Proteome. Nat. Commun. 8, 14357. 10.1038/ncomms14357 28240269 PMC5333359

[B50] Suhre K. McCarthy M. I. Schwenk J. M. (2021). Genetics Meets Proteomics: Perspectives for Large Population-Based Studies. Nat. Rev. Genet. 22 (1), 19–37. 10.1038/s41576-020-0268-2 32860016

[B51] Sun B. B. Maranville J. C. Peters J. E. Stacey D. Staley J. R. Blackshaw J. (2018). Genomic Atlas of the Human Plasma Proteome. Nature 558 (7708), 73–79. 10.1038/s41586-018-0175-2 29875488 PMC6697541

[B52] Vermeulen J. M. Wootton R. E. Treur J. L. Sallis H. M. Jones H. J. Zammit S. (2021). Smoking and the Risk for Bipolar Disorder: Evidence from a Bidirectional Mendelian Randomisation Study. Br. J. Psychiatry 218 (2), 88–94. 10.1192/bjp.2019.202 31526406

[B53] Wind A. E. Takken T. Helders P. J. M. Engelbert R. H. H. (2010). Is Grip Strength a Predictor for Total Muscle Strength in Healthy Children, Adolescents, and Young Adults? Eur. J. Pediatr. 169 (3), 281–287. 10.1007/s00431-009-1010-4 19526369

[B54] Xu H. Gan C. Gao Z. Huang Y. Wu S. Zhang D. (2020). Caffeine Targets SIRT3 to Enhance SOD2 Activity in Mitochondria. Front. Cell Dev. Biol. 8, 822. 10.3389/fcell.2020.00822 33015038 PMC7493682

[B55] Yang J. Fan Y. Yan B. Zhao B. Qian L. Gao F. (2022). Mendelian Randomization Analyses Reveal Novel Drug Targets for Anorexia Nervosa. Prog. Neuropsychopharmacol. Biol. Psychiatry 112, 110427. 10.1016/j.pnpbp.2021.110427 34389437

[B56] Zhao Q. Wang J. Hemani G. Bowden J. Small D. (2020). Statistical Inference in Two-Sample Summary Data Mendelian Randomization Using Robust Adjusted Profile Score. Ann. Stat. 48 (3), 1742–1769. 10.1214/19-AOS1866

[B57] Zheng J. Haberland V. Baird D. Walker V. Haycock P. C. Hurle M. R. (2020). Phenome-Wide Mendelian Randomization Mapping the Influence of the Plasma Proteome on Complex Diseases. Nat. Genet. 52 (10), 1122–1131. 10.1038/s41588-020-0682-6 32895551 PMC7610464

[B58] Zhou W. Nielsen J. B. Fritsche L. G. Dey R. Gabrielsen M. E. Wolford B. N. (2018). Efficiently Controlling for Case-Control Imbalance and Sample Relatedness in Large-Scale Genetic Association Studies. Nat. Genet. 50 (9), 1335–1341. 10.1038/s41588-018-0184-y 30104761 PMC6119127

